# The Association between Mycobacterium Tuberculosis Genotype and Drug Resistance in Peru

**DOI:** 10.1371/journal.pone.0126271

**Published:** 2015-05-18

**Authors:** Louis Grandjean, Tomotada Iwamoto, Anna Lithgow, Robert H Gilman, Kentaro Arikawa, Noriko Nakanishi, Laura Martin, Edith Castillo, Valentina Alarcon, Jorge Coronel, Walter Solano, Minoo Aminian, Claudia Guezala, Nalin Rastogi, David Couvin, Patricia Sheen, Mirko Zimic, David AJ Moore

**Affiliations:** 1 Wellcome Centre for Clinical Tropical Medicine, Imperial College London, St Mary's Campus, Norfolk Place, London, United Kingdom; 2 London School of Hygiene and Tropical Medicine, TB Centre and Department of Clinical Research, Keppel St., London, United Kingdom; 3 Laboratorio de Investigacion y Desarrollo, Universidad Peruana Cayetano Heredia, San Martin de Porres, Lima, Peru; 4 Department of Infectious Diseases, Kobe Institute of Health, Chuo-ku, Kobe, Japan; 5 Johns Hopkins Bloomberg School of Public Health, Baltimore, Maryland, United States of America; 6 DIRESA de Callao, Bellavista, Callao, Peru; 7 Unidad Tecnica TB-MDR, Lima, Peru; 8 TB-Insight Research Group, Rensselaer Polytechnic Institute, Troy, New York, United States of America; 9 Naval Medical Research Unit 6—Bellavista, Callao, Peru; 10 WHO Supranational TB Reference Laboratory, Institut Pasteur de la Guadeloupe, Abymes, Guadeloupe, France; University of Padova, Medical School, ITALY

## Abstract

**Background:**

The comparison of Mycobacterium tuberculosis bacterial genotypes with phenotypic, demographic, geospatial and clinical data improves our understanding of how strain lineage influences the development of drug-resistance and the spread of tuberculosis.

**Methods:**

To investigate the association of *Mycobacterium tuberculosis* bacterial genotype with drug-resistance. Drug susceptibility testing together with genotyping using both 15-loci MIRU-typing and spoligotyping, was performed on 2,139 culture positive isolates, each from a different patient in Lima, Peru. Demographic, geospatial and socio-economic data were collected using questionnaires, global positioning equipment and the latest national census.

**Results:**

The Latin American Mediterranean (LAM) clade (OR 2.4, p<0.001) was significantly associated with drug-resistance and alone accounted for more than half of all drug resistance in the region. Previously treated patients, prisoners and genetically clustered cases were also significantly associated with drug-resistance (OR's 2.5, 2.4 and 1.8, p<0.001, p<0.05, p<0.001 respectively).

**Conclusions:**

Tuberculosis disease caused by the LAM clade was more likely to be drug resistant independent of important clinical, genetic and socio-economic confounding factors. Explanations for this include; the preferential co-evolution of LAM strains in a Latin American population, a LAM strain bacterial genetic background that favors drug-resistance or the "founder effect" from pre-existing LAM strains disproportionately exposed to drugs.

## Introduction

The *Mycobacterium tuberculosis* Beijing clade is thought to be hyper-virulent [1,2] and in some regions has been associated with drug resistance [3]. One of the explanations for this association is the hypothesis that Beijing strains mutate more rapidly than other strains such as those of the Euro-American lineage (lineage 4) [4]. However, recent evidence for the rapid mutation rate of Beijing strains was derived from the comparison of Beijing strains against lineage 4 strains primarily of laboratory origin (CDC 1551, H37Rv and Erdman). Although two clinical strains were also included in this analysis these strains can not necessarily be regarded as representative of the broad diversity within lineage 4 [4].

The association of Beijing strains (or indeed any other strain) with drug resistance could also be biased by a ‘founder effect’. In the Eastern ex-Soviet states where MDRTB is particularly prevalent, strains of the *M*. *tuberculosis* Beijing family have always been dominant while Latin American Mediterranean strains of the Euro-American lineage are under-represented [5,6]. When the national tuberculosis programs of the ex-Soviet Union failed, Beijing strains were disproportionally subjected to failing treatment regimens as well as being exposed to poor treatment within the prison system [7].

The Latin American Mediterranean family has been associated with drug resistance in sub-population level studies in Kwa-Zulu Natal [8], Brazil [9], Russia [10] and the Ukraine [6]. The Latin American Mediterranean family and the Haarlem family both of the Euro-American lineage are dominant in South America. However, the country of Peru has both Latin American Mediterranean strains, Haarlem strains and the highest proportion of Beijing strains in the Americas [11]. Peruvian Beijing strains were imported to the country well before the advent of antibiotics in the mid 19^th^ century along with significant Chinese migration to the country [12]. Beijing, Haarlem and Latin American strains in Peru were then subjected to an inadequate national TB program in the 1980’s and early 1990’s [13] which preceded a dramatic rise in drug resistance.

To determine which *M*. *tuberculosis* genotypes were most associated with drug resistance, we undertook a prospective population level molecular epidemiological study of incident tuberculosis cases in Lima, Peru.

## Methods

### Field Methods

Between January 2008 and February 2010, the MODS assay [14–16] for rapid, liquid culture diagnosis of TB and MDRTB was implemented in Lima South (population 1,455,946 [17]) and Callao (population 876,877 [17]), two of the four large regions of metropolitan Lima (S1 Fig). Integrated into the national tuberculosis program, the test was made available for free, to all patients with symptoms of tuberculosis in both regions. A pro forma detailing previous treatment history, address, sex and age was completed by the health post or hospital when requesting a MODS test for all new symptomatic patients. All hospitals and clinics in both regions sent samples to the only reference laboratory in each region where the MODS test was performed.

### Laboratory Methods

Strains from positive MODS liquid cultures were sub-cultured onto solid Ogawa medium and transported to the research laboratory at Universidad Peruana Cayetano Heredia (Lima, Peru) for DNA extraction [18] and spacer-oligonucleotide typing (‘spoligotyping’) as described previously [19]. Automated 15-loci MIRU-VNTR was performed at the Kobe Institute (Kobe, Japan) following established protocols [20]. The denominator of all culture positive sputum samples sent to both regional laboratories was obtained from the national reference laboratory database. All drug resistant strains were sent to the national reference laboratory to confirm the diagnosis of drug resistance by the proportions method and a subset of half of all drug resistant samples were sent for illumina high-seq sequencing to confirm the diagnosis by molecular methods.

Strains were named by uploading the combined MIRU and spoligotype data to the MIRU-VNTRplus website (www.miru-vntrplus.org) and following their protocol. Strains that could not be named because they did not match a clade in the MIRU-VNTRplus database were termed ‘unknown’ strains. Any strains that failed spoligotyping or MIRU-VNTR (because one or more loci were ambiguous by either MIRU or spoligotyping) were also genotyped again by both techniques, if they failed a second time they were excluded from the analysis.

### Spatial and Statistical Analysis

Socio-economic status per city block (upper, middle and lower tertile) and population density (number of people per city block) was obtained from the latest Peruvian National Census [21]. All cases that could be mapped, were mapped to their place of residence at the level of the city block either manually with a handheld GPS machine or where possible directly onto Google Maps using Google Earth 9.0. Mapped cases were combined with census data by spatially merging the geographic coordinates using ArcGIS.

Data was analyzed in ‘R’ (R Foundation for Statistical Computing, Vienna, Austria 2011, www.R-project.org) and Stata (Release 11, StataCorp. 2009). The Manhattan distance (the sum of all absolute pair-wise differences between loci) was used to determine the genetic distance between genotypes. Minimum spanning trees were constructed using Cytoscape rather than Bionumerics in order to present the data more effectively using the organic graph drawing algorithm in Cytoscape. The UPGMA (Unweighted Paired Group Method with Arithmetic Mean) algorithm was chosen to construct the phylogeny because it allowed us to make a like for like comparison of our phylogeny with the phylogeny of the strain collection of the MIRU-VNTRplus website.

Determinants of drug resistance (any drug resistance vs drug susceptibility) were tested in the context of a multivariate logistic regression. All predictor variables with a significance of p<0.2 on univariate analysis and any potentially important confounding variables were included in a multivariate logistic regression analysis. All biologically plausible interactions were also examined for significance in the model. A significance value of p<0.05 was chosen for predictor variables in the multivariate model. The Haarlem clade was chosen as the reference comparison for all other clades as it was the second most prevalent clade in the population. The model was also re-run with a dichotomized clade variable (e.g. Beijing vs non-Beijing) to make comparisons between the clade in question and all other clades.

### Independent Data Set Comparison

The principal study outcome was compared to an independent dataset compiled by the Institut Pasteur Guadaloupe [22] of 2192 different strains collected across South America all of which had the strain genotype and phenotype available for comparison. The study dataset was also independently analyzed by DC and NR at the Institut Pasteur Guadeloupe.

### Ethics Statement

Ethical approval was obtained from the institutional review board of Universidad Peruana Cayetano Heredia before the study began. Consent was not obtained from participants because the data were analyzed anonymously after checking for duplicated patients. Institutional approval for the study was obtained from the Peruvian Ministry of Health.

## Results

### Study Recruitment

The first positive culture of 2,139 different tuberculosis patients (2,139 cultures from 2,139 patients) was genotyped by both 15-loci MIRU-VNTR and spoligotyping. A total of 2086 strains were successfully genotyped (53 genotypes were excluded because either MIRU typing or spoligotyping failed or generated ambiguous results) and formed the core of the analysis. The demographic data of these patients is given in Table 1. A total of 1825/2086 (87% of genotyped patients) could be mapped at the level of the city block of which 1698/2,086 (81% of genotyped patients) had data available from the Peruvian National Census 2007. The study was undertaken between January 2008 and February 2010. A coverage of 71.4% of the entire culture positive population in the region was reached for 14 consecutive study months representing 1993 patients from a total of 2790 possible sputum culture positive samples in this time period.

**Table 1:** Demographic Details for All Genotyped Patients included in the Study.

Demographics	Drug Susceptible^1^	MDR	Isoniazid Mono-Resistant	Rifampicin Mono-Resistant	Overall
Number (% of Total)	1684 (81%)	209 (10%)	121 (6%)	52 (2%)	2086 (100%)^†^
Median Age (IQR)	31 (24–44)	30 (25–42)	33 (25–45)	33 (25–40)	31 (24–44)
Sex—Male (%)	1060 (63%)	128 (61%)	72 (60%)	34 (67%)	1304 (63%)
Smear Positive (%)	1487 (89%)	181 (89%)	109 (90%)	46 (88%)	1831 (88%)
HIV Positive (%)	46 (3%)	3 (1%)	2 (2%)	1 (2%)	52 (2.5%)
History of Previous Treatment	384 (23%)	100 (48%)	34 (28%)	24 (46%)	548 (26%)
Upper SES^2^	65 (5%)	8 (5%)	9 (9%)	0 (0%)	82 (5%)
Middle SES^2^	480 (35%)	63 (40%)	34 (32%)	13 (28%)	590 (35%)
Lower SES^2^	842 (60%)	88 (55%)	62 (59%)	34 (72%)	1026 (60%)
Population Density (People per Block) Median (IQR)	130 (74–149)	142 (84–168)	131 (86–167)	130 (86–149)	131 (81–149)

^1^Susceptible to rifampicin and isoniazid.

^2^Average Socio Economic Status (SES) of the city block in which the patient lived.

^†^Phenotypic data was not available for <1% of samples.

### Patient Demographics

Eighteen percent (18%, 95% CI 16.8–20.2) of all patients were resistant to at least one of rifampicin or isoniazid, while 10.1% (95% CI 8.8–11.4) were multidrug-resistant. Two-thirds of patients were male and the median age was 31 years. The percentage of HIV positive patients was 2.5% (95% CI 1.9–3.3) and 88% (95% CI 87.2–90.0) were sputum smear positive. Most tuberculosis patients lived in city blocks in the lower socio-economic tertile (60% overall 95% CI 58.1–62.8). A history of prior tuberculosis treatment was more common in patients with MDRTB (48%, 95% CI 40.9–54.8%) and rifampicin mono-resistance (46%, 95% CI 32.2–60.5%) than amongst patients with drug susceptible disease (23%, 95% CI 20.8–24.9%) (p<0.001 for both, test of proportions), but not in patients with isoniazid mono-resistance (28%, 95% CI 20.3–36.9%, p = 0.2, test of proportions).

Multidrug resistance was detected in 7% (95% CI 5.9–8.6%) of new and 18% (95% CI 15.2–22.0%) of previously treated patients. Sixty six percent (66/100, 95% CI 55.8–75.1%) of MDRTB patients with a previous treatment history shared identical MIRU and spoligotypes with another patient in the study region.

Data on age and sex was available for the remainder of patients not included in the study. The median age (32 years, p = 0.5) and sex (66% males, p = 0.4) was not significantly different from that of included subjects.

### Cluster Analysis and Population Structure

**Fig 1:**
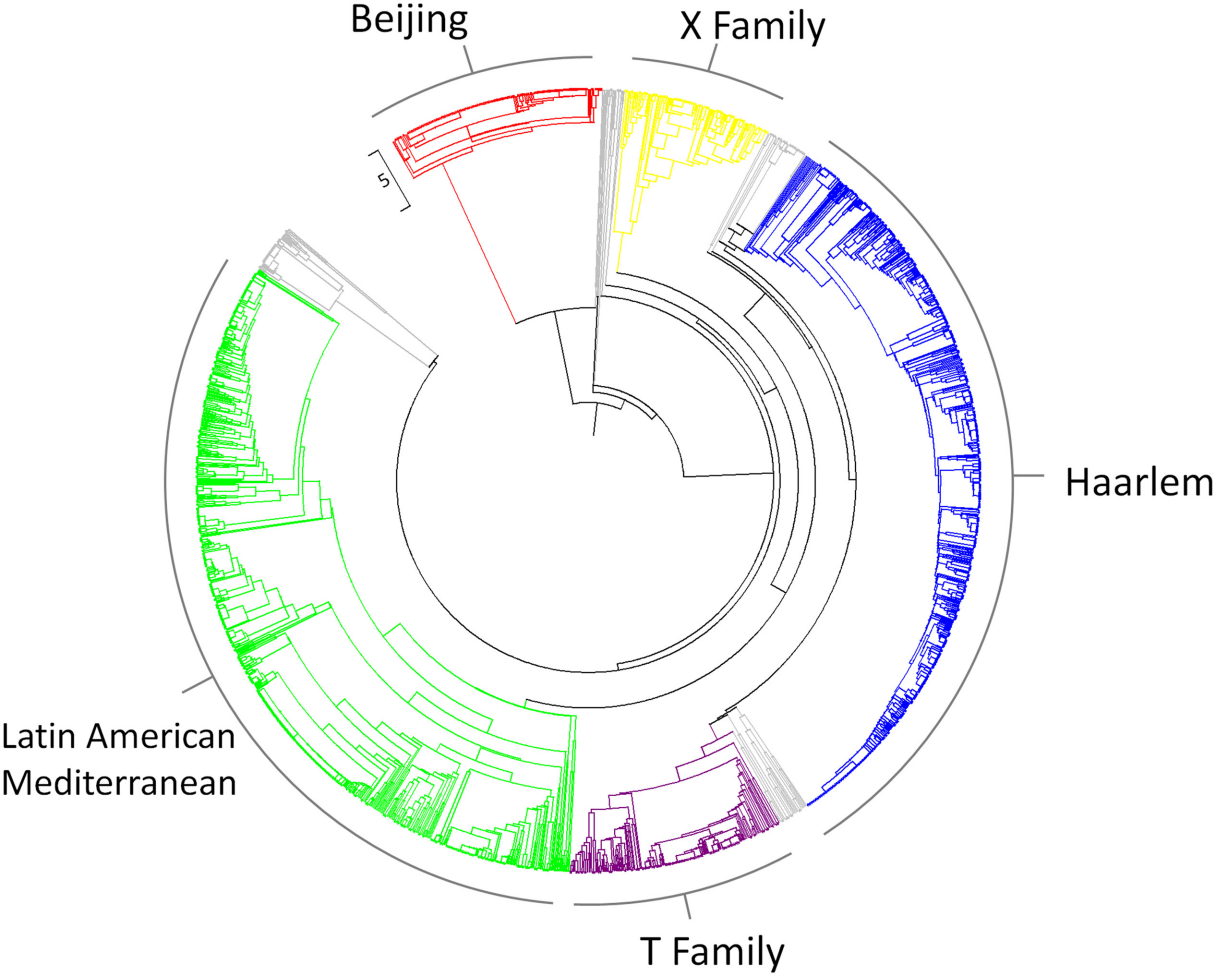
A UPGMA phylogeny of all study strains. Beijing strains coloured red, X clade coloured yellow, Haarlem clade blue, T clade purple and Latin American Mediterranean green. Strains belonging to other families and strains that could not be subscribed a family as they were too distant from strains in the MIRU-VNTRplus database are shown in grey.

The three most frequently observed clades were the LAM (Latin American Mediterranean) clade (736/2086, 35%, 95% CI 33.2–37.4%) and the Haarlem clade (690/2086, 33% 95% CI 31.1–35.1%) which are both part of the Euro-American lineage, and the Beijing clade, (197/2086, 9%, 95% CI 8.2–10.8%) part of the East Asian lineage (Fig 1). Other strains included those from the X family (135/2086, 6.5%, 95% CI 5.4%-7.6%) and T strains (196/2086, 9.4%, 95% CI 8.2–10.7%).

### Association of Genotype with Drug Resistance

**Fig 2:**
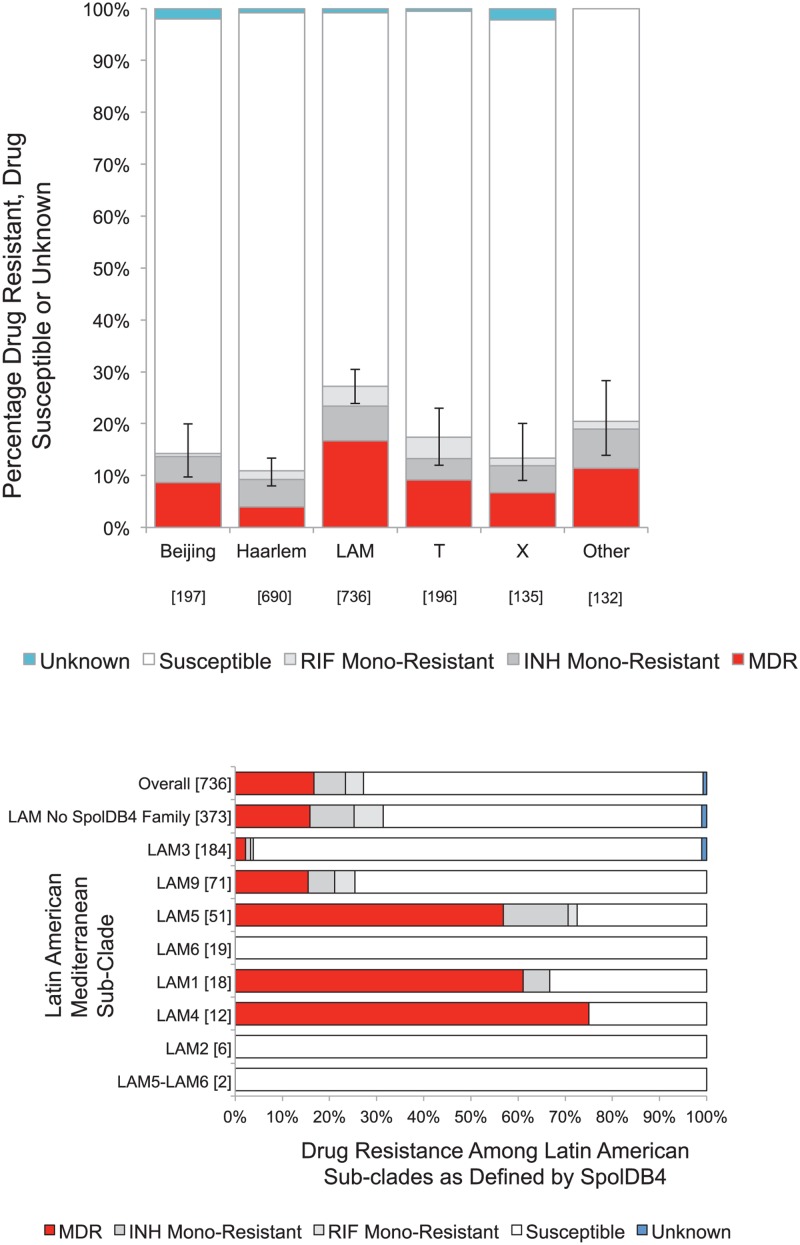
a) Percentage of drug resistance by clade. Numbers are given in square brackets b) Drug resistance within the LAM clade stratified by SpolDB4 designated spoligotype. Numbers are given in square brackets.

The proportion of drug resistance was calculated for each clade (Fig 2a). The LAM clade accounted for 59% (123/209, 95% CI 52.8–65.9%) of all multidrug-resistance, 54% (28/52, 95% CI 39.5–67.8%) of rifampicin mono-resistance and 40% (49/121, 95% CI 31.3–49.1%) of isoniazid mono-resistance. The Haarlem clade accounted for 13% of multidrug-resistant strains (27/209, 95% CI 8.7–18.2%), 21% of rifampicin mono-resistant strains (11/52, 95% CI 11.0%-24.7%) and 31% of isoniazid mono-resistance (37/121, 95% CI 22.5%-39.6%). The Beijing strains accounted for 8% of all multidrug-resistance (17/209, 95% CI 4.8%-12.7%), 2% of rifampicin mono-resistance (1/52, 95% CI 0.05%-10.2%) and 8% of isoniazid mono-resistance (10/121, 95% CI 4.0%-14.7%). Strains from the X-family accounted for 4% of multidrug-resistance (9/209, 95% CI 2.0–8.0%), 4% of rifampicin mono-resistance (2/52, 95% CI 0.5%-13.2%) and 5.8% of isoniazid mono-resistance (7/121 95% CI 2.4–11.6%) while T strains accounted for 8.6% of multidrug-resistance (18/209, 95% CI 5.2%-13.3%), 15% of rifampicin mono-resistance (8/52, 95% CI 6.9–28.1%) and 7% of isoniazid mono-resistance (8/121, 95% CI 2.9–12.6%).

**Fig 3:**
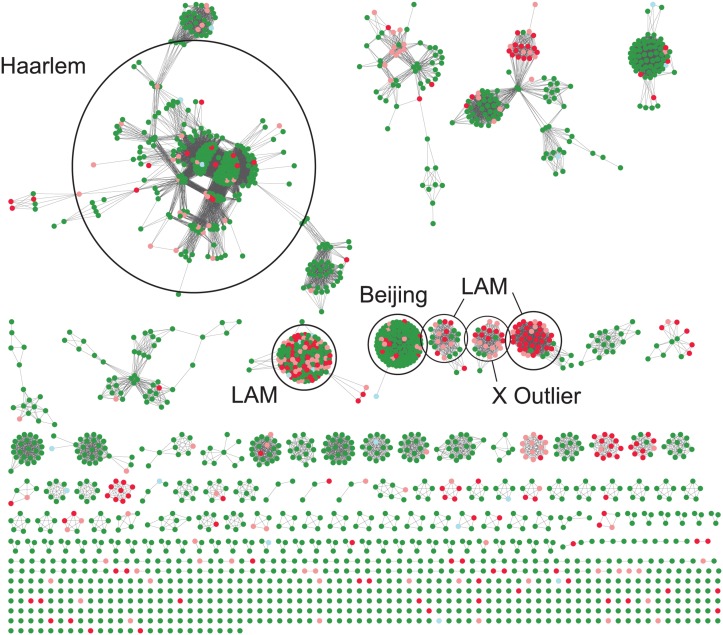
A minimum spanning tree of all study genotypes. Each coloured node is a different patient; green drug sensitive, red multidrug resistant, pink mono-resistant, light blue unknown. Patients grouped closely together have identical MIRU types and spoligotypes. A linking line between nodes denotes a change in Manhattan distance equal to 1.

More than a quarter of the LAM sub-groups LAM1, LAM4, LAM5 and LAM9 (as defined by the SpolDB4 international database) strains were drug resistant. However, not all LAM sub-group strains were associated with drug resistance (Fig 2b). A minimum spanning tree of all 2086 strains coloured according to drug resistance demonstrates the clusters with the highest proportion of drug resistance (Fig 3). Linked nodes that differed by a Manhattan difference of 1 demonstrate possible strain evolution between patients. The largest cluster in the minimum spanning tree was part of the Haarlem clade. Both clusters and unique strains that were part of the LAM clades had the highest proportions of drug resistance and the Beijing clade demonstrated a high level of clonality.

### Predictors of Drug Resistance

Multivariate logistic regression demonstrated that the LAM clade (OR 2.4, 95% CI 1.8–3.0, p<0.001) was more likely to be drug resistant than all other clades. Neither the Beijing clade (OR 0.6, 95%CI 0.4–0.9, p = 0.03), the T clade (OR 1.1, 95% CI 0.7–1.7, p = 0.69), the Haarlem clade (OR 0.5, 95% CI 0.3–0.6, p<0.001) nor the X clade (OR 0.7, 95% CI 0.4–1.2, p = 0.28) were associated with drug resistance relative to all other clades. A history of previous treatment also increased the odds of drug resistance by 2.5 fold (OR 2.5, 95% CI 1.9–3.3, p<0.001) as well as being imprisoned (OR 2.4, 95% CI 1.1–5.2, p<0.001). Patients who were a member of a genetic cluster were 1.8 times more likely to have drug resistance (OR 1.8, 95% CI 1.4–2.4, p<0.001). No other factors were significantly associated with drug-resistance (Table 2).

**Table 2:** The Predictors of Drug Resistance (multivariate logistic regression analysis).

	Association with Drug Resistance^1^		
Predictor	Odds Ratio	95% CI	p-Value
Age	0.9	0.9–1.0	0.20
Sex (Male)	0.9	0.6–1.1	0.29
HIV Positive	0.6	0.2–1.5	0.24
Suptum Smear Positive	1.2	0.8–1.9	0.42
Previously Treated	2.5	1.9–3.3	<0.001
Haarlem Clade	0.5	0.3–0.6	<0.001
LAM Clade	2.4	1.8–3.1	<0.001
Beijing Clade	0.6	0.4–0.9	0.03
T Family Clade	1.1	0.7–1.7	0.69
X Clade	0.7	0.4–1.2	0.28
Other	0.6	0.2–1.9	0.35
Population Density	1.0	0.9–1.0	0.22
Upper SES3 (comparison level)^3^	-	-	-
Middle SES3^3^	0.9	0.5–1.7	0.74
Lower SES3^3^	0.8	0.5–1.6	0.61
Imprisoned	2.4	1.1–5.2	0.03
Part of a Genetic Cluster^2^	1.8	1.4–2.4	<0.001

^1^Resistance to rifampicin, isoniazid or both.

^2^Genetic Clustering defined as identical 15 loci MIRU type and identical spoligotype.

^3^Average Socio Economic Status of the city block in which the patient lived (SES), comparison was made to Upper SES as a reference.

### Independent Data Set Comparison

The independent dataset compiled by the Institut Pasteur Guadaloupe contained a total of 2192 different strains collected from diverse locations in South America together with their drug resistance phenotype and genotype. These 2192 strains were isolated in Peru (n = 794), Brazil (n = 286), Colombia (n = 519), French Guiana (n = 268), Guyana (n = 90), Surinam (n = 102), Venezuela (n = 131) and Bolivia (n = 2). Latin American Mediterranean strains comprised 32.5% (713/2192) of the dataset. Thirty percent (218/713) of LAM strains were drug resistant, while 25% of strains from other lineages were drug resistant (376/1479). Latin American strains again had a significantly higher proportion of drug resistance in this independent dataset (p = 0.01, Two-tailed test of proportions). An independent analysis of the 2086 strains in the study dataset undertaken by DC and NR at the Institut Pasteur confirmed the study findings.

## Discussion

This large prospective molecular epidemiological study of tuberculosis conducted in a high incidence multidrug-resistant tuberculosis setting demonstrates a significant clade specific association with drug resistance independent of genetic clustering, and important clinical and socioeconomic confounding factors. The Latin American Mediterranean (LAM) clade was highly associated with drug resistance while the Haarlem and Beijing strains were less likely to be drug resistant.

The founder effect could explain the association of LAM strains with drug resistance in South America and the association of Beijing strains with drug resistance in Asia [23,24]. However, in Peru because of European and Asian immigration a diversity of strains existed that predated the use of antibiotics. Strains from the Haarlem, Beijing and Latin American Mediterranean families were very likely to have been circulating in the population for over a century prior the use of antibiotics, yet only the Latin American Mediterranean family has emerged as being highly associated with drug resistance. The association of these strains with drug resistance remained statistically significant in both clustered and non-clustered strains excluding the role of a purely clonal expansion of drug resistant strains. One possible explanation for our finding is that LAM strains could harbor advantageous mutations that allow them to maintain fitness while also becoming drug resistant. Differential host-pathogen co-evolution may also explain why Latin American strains are so associated with drug resistance in South America. Latin American strains may more easily become drug resistant within a Latin American host. The prison within the study area had a prevalence of Beijing strains that was three times that of the surrounding community and prisoners were twice as likely to be drug resistant which suggests that the prison could act as an amplifier of drug resistant Beijing strain transmission in the community.

A sub-population level study of drug resistant strains in South Africa suggested that the LAM4 clade is one of the main contributors to the extensively drug resistant (XDR) tuberculosis outbreak [8]. This association has also recently been described in another study of 237 isolates in Brazil that also lacked the denominator of all strains to be able to compare the proportion of drug resistance between clades [9]. Interestingly the LAM subtypes most associated with drug resistance in the Brazilian study were the LAM1, LAM4, LAM5 and LAM9 strains, exactly the same subtypes that had the highest levels of drug resistance in our data set. Pre-existing evidence of this association in other regions and our confirmation of the association in an independent dataset makes our findings relevant across South America. One previous study undertaken in Peru by our research group with an independent set of hospital derived strains also highlighted the association of LAM9 with MDRTB [25], while another study by Taype et al supports our finding that Beijing strains are not associated with drug resistance in Lima [26].

This study benefited from the population level implementation of a sensitive and inexpensive diagnostic test, which enabled data to be gathered on a large proportion of new tuberculosis cases in a study area of approximately 2.3 million people. However, whilst epidemiological links are not necessary for tuberculosis to be transmitted [27] the knowledge of epidemiological links between cases would have improved our cluster definition. The duration of high population coverage in this study was 14 months, a longer duration of high coverage would limit the contribution of the strains diagnosed at the beginning and the end of the study and ensure that saturation of the clustered proportion was reached [28].

It is also necessary to acknowledge the limitations of MIRU-typing and spoligotyping. These techniques do not provide the same level of phylogenetic quality and resolution as 24-loci MIRU typing, whole genome sequencing and long sequence polymorphism typing. This risks misclassification of some strains into clades that are closely related by MIRU-typing and spoligotyping but determined to be more distantly related by whole genome sequencing or long sequence polymorphism typing. However, despite the limitations, MIRU-VNTR has been demonstrated to generate phylogenetic relationships that are broadly congruent with those of SNP typing, spoligotyping and long sequence polymorphisms [29]. MIRU-VNTR is still widely used for population level molecular epidemiological genotyping [20] and the resolution provided by MIRU-typing is also maximized in strains of the Euro-American lineage most frequently observed in our data set [30].

This population level study of tuberculosis in Lima has identified the Latin American Mediterranean clade as being highly associated with a drug resistant phenotype. Patients with drug sensitive disease caused by LAM strains may be more likely to acquire drug resistance or cause secondary cases of drug resistant disease after having become drug resistant. The standard Directly Observed Therapy short course (DOTs) is failing to prevent the onset and spread of drug resistant tuberculosis, particularly among these patients. In settings where LAM1, LAM4, LAM5 and LAM9 strains are highly prevalent it is particularly important that robust mechanisms are in place for drug resistance testing and surveillance.

## Supporting Information

S1 Fig A map of metropolitan Lima with the study area shaded in yellow. The region of Callao is to the North and Lima South to the South.
(TIF)
