# Supplementary figures and images for: The Association between Mycobacterium Tuberculosis Genotype and Drug Resistance in Peru
Source: PLoS One. 2015 May 18;10(5):e0126271. doi: 10.1371/journal.pone.0126271 (PMC4435908; doi:10.1371/journal.pone.0126271)

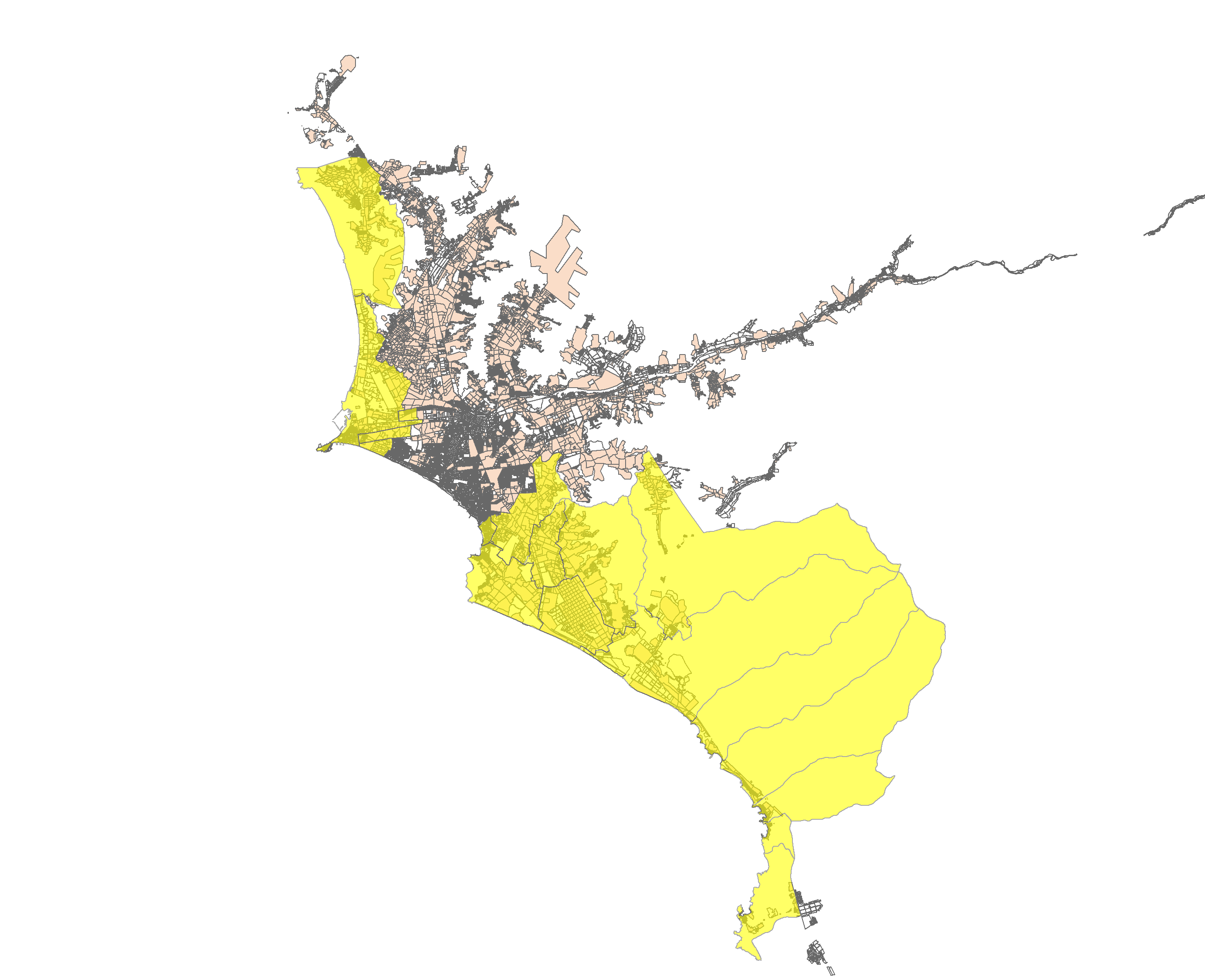

Supplement: S1 Fig — The region of Callao is to the North and Lima South to the South. (TIF) [file pone.0126271.s001.tif]
